# Myelofibrosis-Related Arthritis Successfully Treated with Hydroxyurea

**DOI:** 10.1155/2014/869743

**Published:** 2014-04-10

**Authors:** Xavier Guillot, Marius Moldovan, Claire Vidon, Daniel Wendling

**Affiliations:** ^1^Department of Rheumatology, Besançon University Hospital, boulevard Fleming, 25030 Besançon Cedex, France; ^2^Department of Internal Medicine, Belfort-Montbéliard Hospital, 14 rue de Mulhouse, 90000 Belfort, France

## Abstract

A 62-year-old woman suffering from one-year lasting, nonerosive peripheral arthritides with general health impairment and high acute-phase reactant levels was admitted to rheumatology department. The patient had suffered from chronic polyarthralgia and a thrombocytosis had been discovered 9 years before, with a recent increase in platelet count. All immunological blood tests were negative. Corticosteroid and methotrexate treatments improved pain, swollen joint count, and systemic inflammation. However, joints remained stiff and painful with two swollen wrists and persistent thrombocytosis. An iliac bone marrow biopsy was performed, showing primary myelofibrosis. Hydroxyurea treatment (500 mg per day) allowed to achieve complete and prolonged clinical and biological remission. After 6 months, a new disease flare occurred. The patient reached remission again after hydroxyurea dose increased to 1500 mg per day. This supports the hypothesis of idiopathic myelofibrosis-associated seronegative polyarthritis. This is the first reported case in which haemopathy-targeted treatment using hydroxyurea induced arthritis remission.

## 1. Introduction

A 62-year-old woman with one-year lasting peripheral arthritides, Raynaud phenomenon, sclerodactyly, and severe global health impairment was admitted to rheumatology department. This patient had been treated by hydroxychloroquine for 13 years for seronegative polyarthralgia. A chronic progressive JAK2-negative thrombocytosis had been discovered 9 years before, considered as essential thrombocythemia.

## 2. Case Presentation

**Figure 1:**
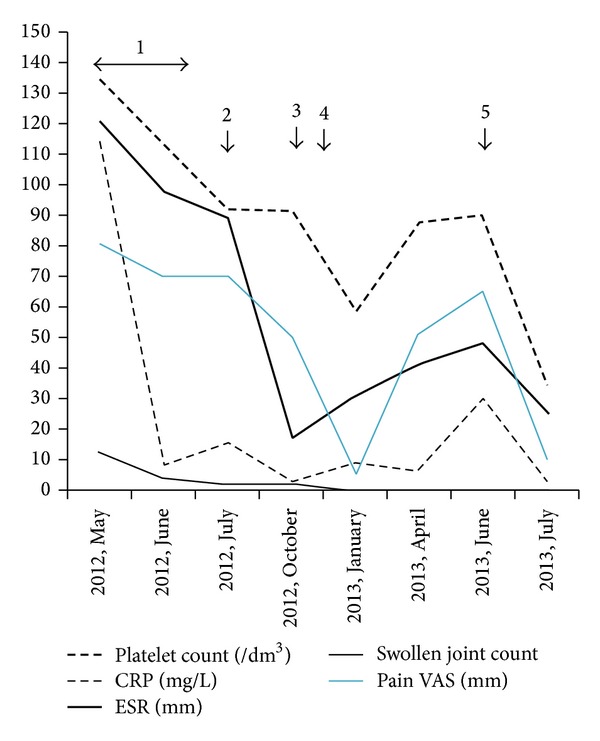
Clinical and biological parameter evolution under treatments. (1) Methylprednisolone 120 mg : 3 infusions, (2) methotrexate 10 then 15 mg a week (4 months) + prednisone 10 mg, (3) hydroxyurea 500 mg daily + degressive prednisone 40 mg → 5 mg (1 month), (4) corticosteroid stop, and (5) hydroxyurea 1500 mg daily, no corticosteroid.

A recent increase in platelet count was attributed to a systemic inflammation that appeared a few months before. The patient had 12 swollen joints, no organomegaly nor clinical lymph node enlargement. Biological tests showed 1340000 platelets per mm^3^, hemoglobin 7.5 g/dL with inflammatory profile, leukocytes 8300 per mm^3^, neutrophils 6931 per mm^3^, lymphocytes 830 per mm^3^, 109 myelocytes per mm^3^, CRP 114 mg/L, and ESR 120 mm. Immunological tests, plasmatic protein electrophoresis, salivary gland biopsy, joint X-rays, and positron emission tomography were not contributive. Three methylprednisolone 120 mg infusions, followed by oral daily 10 mg prednisone, improved pain, swollen joint count, and acute-phase reactant levels (CRP 2.9 mg per liter, ESR 17 mm after 1 hour). Wrists remained painful and slightly swollen with persistent hand joint stiffness in spite of methotrexate treatment (10 then 20 mg a week for 4 months). As platelets remained high (930000 per mm^3^) even after systemic inflammation decrease, an iliac bone marrow biopsy was performed, revealing hyperplasic megakaryocytic (with hypertrophic, slightly dystrophic nuclei) and granulocytic lines, moderately hypoplastic erythroblastic line, focal lymphoid aggregate, and moderate reticulin fibrosis. Immunohistochemical analysis showed no abnormality. Idiopathic myelofibrosis was diagnosed. After collegial discussion, methotrexate was stopped and a treatment by hydroxyurea (500 mg daily) and prednisone (40 mg degressive over 1 month) was started. After 2 months and for the first time, the patient reached complete clinical and biological (acute phase reactants, hemogram) remission, which was confirmed after 6 months. At this point, platelet levels reascended as well as patient's pain VAS and CRP levels. However, there was no swollen joint. Hydroxyurea dose increase (1500 mg daily) without any corticosteroid treatment normalized pain VAS, ESR, and CRP levels, which was still the case 1 month later, as shown in Figure 1. This could support the diagnostic hypothesis of idiopathic myelofibrosis-associated seronegative polyarthritis.

## 3. Discussion

This entity has already been described before [1]. Myelofibrosis is a myeloproliferative syndrome which may be idiopathic or associated with autoimmune diseases such as systemic lupus erythematosus [2], Sjögren's syndrome [3, 4], progressive systemic sclerosis [5], and rheumatoid arthritis [6–8]. In case of primary myelofibrosis, seronegative arthritis may be associated [9]. Hydroxyurea could have modest therapeutic effects in rheumatoid arthritis [10]; however disease remission observed in our patient is more likely related to primary hematologic disease treatment, as remaining joint symptoms and thrombocytosis recovered concurrently. Thrombocytosis is a haematologic feature which may be related to systemic inflammation in arthritic patients. However, in this case, platelet levels remained high in spite of ESR and CRP normalization (Figure 1). This highlights the importance of investigating haemogram abnormalities with inflammation-disconnected evolution before starting any biologic treatment, in order not to miss a potential myeloproliferative syndrome. Whether myelofibrosis is idiopathic or secondary to the joint disease is controversial. Thrombocytosis could also have been secondary to systemic inflammation. However, platelet count remained high despite inflammation decrease before specific haematologic disease-targeted treatment. Furthermore, treatment with hydroxyurea allowed to achieve long-term arthritis remission (after dose adjustment), which had not been the case with corticosteroids alone. Such an evolution had never been reported before. This could support the hypothesis of idiopathic myelofibrosis-associated seronegative polyarthritis, regressive after low-dose hydroxyurea treatment.
